# The Biological Functions and Mode of Action of Transcription Factor ELF4: A Promising Target for Treating Intestinal Homeostasis Disorder-Related Diseases

**DOI:** 10.3390/biology14111480

**Published:** 2025-10-23

**Authors:** Linjiang Xie, Haixin Bai, Ziyi Bai, Lv Fu, Haitao Yu

**Affiliations:** 1 State Key Laboratory of Animal Nutrition and Feeding, Ministry of Agriculture and Rural Affairs Feed Industry Centre, China Agricultural University, Beijing 100193, China; xielinjiang@cau.edu.cn (L.X.); b20243040451@cau.edu.cn (H.B.); sy20243041045@cau.edu.cn (Z.B.); 2 Frontier Technology Research Institute of China Agricultural University in Shenzhen, Shenzhen 518119, China; 3 Key Laboratory of Animal Genetics, Breeding and Reproduction in the Plateau Mountainous Region, Ministry of Education, College of Animal Science, Guizhou University, Guiyang 550025, China; gs.lfu23@gzu.edu.cn

**Keywords:** transcription factor ELF4, immune regulation, intestinal inflammatory-related disease, barrier function, gut microbiota, pathogen infection

## Abstract

**Simple Summary:**

Dysregulated intestinal homeostasis is a major global health threat, affecting an estimated 1–1.5 billion people worldwide with conditions like inflammatory bowel disease, chronic diarrhea, and dysmotility. The core mechanisms involve gut dysbiosis, immune dysfunction, and impaired epithelial barrier function. Targeting transcription factors, key regulators of gene expression, offers a promising strategy for precision medicine. This review synthesizes evidence highlighting epithelial transcription factor ELF4 as a crucial host protective factor. Research has demonstrated ELF4’s significant roles in maintaining intestinal barrier integrity, promoting microbial balance, modulating inflammatory responses in the gut, and enhancing anti-infection defenses. Thus, ELF4 emerges as a novel therapeutic target for restoring intestinal homeostasis and enabling precision treatment. Future research into ELF4 and its downstream pathways will further elucidate the molecular basis of intestinal diseases, paving the way for more effective therapies and offering new hope for patients suffering from these prevalent disorders.

**Abstract:**

Intestinal homeostasis disorders (IHDs), driven by food safety issues, pollution, and drug-resistant pathogens, threaten global health. Key factors in intestinal and metabolic diseases (like IBD, obesity, and liver disease) include barrier dysfunction, gut microbiota imbalance, and excessive immune activation. Transcription factors in intestinal epithelial cells are crucial regulators. ELF4, an ETS family transcription factor, plays vital roles in transcriptional regulation, impacting tumorigenesis, the DNA damage response, and the cell cycle. ELF4 deficiency exacerbates alcoholic liver disease (ALD). Significantly, ELF4 is a novel IFN-I transcription factor with antiviral capabilities. Its regulation of the type I IFN response offers new avenues for developing antiviral and anticancer strategies and managing IFN-induced autoimmune disorders. Thus, ELF4 emerges as a promising target for preventing and treating IHD-related diseases. Mechanistic studies could help identify diets or antimicrobials that activate intestinal ELF4 to combat pathogen/virus-induced intestinal diseases.

## 1. Introduction

Bacterial resistance and reducing antibiotic dependence are important issues of common concern in the fields of human medicine and animal health care. In the post-antibiotic era, the increase in intestinal diseases and mortality has become one of the world’s most serious public health problems. Additionally, there has been a lag in research on and development of new antibiotics [1,2,3,4,5], accompanied by an increase in pathogenic organisms’ pathogenicity and resistance [6,7,8], a decrease in host immune function and remodeling ability, and frequent occurrence of intestinal opportunistic infections [9,10,11,12,13,14,15,16,17,18,19]. Most strategies for defending against pathogen infections can be divided into one of two categories: controlling and killing pathogens or strengthening host immunity. Pathogen control aims to curb the spread of pathogens through prevention. In strengthening host immunity, both studying the host’s antipathogen immune mechanism and developing anti-infective drugs are important to control pathogen infection in clinical treatment. Notably, innate immunity is critical to the recognition of infection by pathogenic organisms and the rapid response to infection [20,21,22,23,24,25,26,27]; thus, it is indispensable to the prevention of infections and the maintenance of immune balance and body health.

E74-like Factor 4 (ELF4) is a novel interferon (IFN)-I transcription factor (TF). The discovery that ELF4 regulates the type I IFN response provides new insight, methods, and perspectives in the development of antiviral and anticancer strategies and treatment of autoimmune disorders induced by IFNs [28,29]. In view of ELF4’s role in immune regulation, it could become a focus in future studies on both infectious and noninfectious diseases. In addition, ELF4 is involved in various physiological and pathological cell processes, such as tumorigenesis [30], the DNA damage response [31], and cell cycle regulation [32]. Although there have been an increasing number of studies in these areas, the exact biological function of ELF4 remains elusive. In recent years, the study of the functional relationship of genes in intestinal homeostasis disorder (IHD)-related diseases has become a research hotspot, which has deepened the understanding of the genetic basis of rare diseases. It has also raised new questions; for example, the study of monogenic autoinflammatory diseases may involve the discovery of genes and effective pathways that regulate human inflammation, which could facilitate the discovery of new targets [33]. Moreover, relevant preclinical and clinical studies have shown that intestinal homeostasis dysfunction, especially intestinal barrier dysfunction and the imbalance of immune and microbial homeostasis, is an important factor in the pathogenesis of many diseases, such as intestinal inflammatory diseases (IBDs) and metabolic disorders (MetS), including liver cirrhosis, inflammatory bowel disease, rheumatoid arthritis, psoriasis, Parkinson’s disease, and autism, which are prevalent in a variety of patients [34,35,36,37]. The intestinal mucosal system performs an important physiological function in maintaining the ecological balance of the intestinal tract. The intestinal mucosa can protect the host by preventing the invasion of pathogenic microorganisms and toxins in the intestinal lumen. It is mainly composed of the intestinal physical barrier, chemical barrier, immune barrier, and microbial barrier. These four intact barriers are organic and function together through a variety of cells and signaling pathways to perform different biological functions and maintain intestinal homeostasis [38,39,40]. Intestinal epithelial cells utilize a variety of strategies to resist bacterial adhesion and invasion, including the secretion of antibacterial peptides, mucus construction, tight junction formation, and innate pathogen sensing. Intestinal epithelial cells and the innate immune system in the intestine communicate with each other to form a functional barrier against antigens, including various pathogens. When pathogens invade, the innate immune system can rapidly mobilize innate immune components to fight the pathogens, confining them to the site of invasion. Additionally, intestinal symbionts strengthen the intestinal barrier through direct and indirect mechanisms, thereby promoting host health. Therefore, the intestinal epithelium regulates the development and colonization of the microbiota, while commensal bacteria, in turn, regulate the mucosal immune response. Moreover, they are interdependent and interact with the gut microbiota to jointly regulate the host’s physiological functions [41,42,43,44,45]. Intestinal destruction is often a critical factor in diseases related to IHDs. In addition, intestinal barrier dysfunction caused by genetic factors could further lead to changes in gut microbiota and cause pathogen-induced inflammation. For instance, glucagon-like peptide-2 (GLP-2) is an intestinal epithelial-specific growth factor that promotes the absorption of nutrients in the intestinal tract and maintains the normal function of the intestinal barrier. It also plays a role in intestinal mucosal repair [46]. For example, previously published studies have reported that intestinal flora disorders destroy GLP-2’s intestinal barrier maintenance function, thereby disrupting the intestinal barrier [47]; reduced beta Klotho (KLB) expression caused by gene mutations is associated with increased intestinal permeability in patients with diarrhea-predominant irritable bowel syndrome. Furthermore, KLB forms complexes with tight junction proteins (TJPs) in intestinal epithelial cells to prevent alcohol-induced endocytosis and degradation of TJPs, protect the intestinal barrier, and improve alcoholic liver disease [48]. Intestinal HIF-1α is critical to the adaptive response of alcohol exposure-induced changes in gut microbiota and barrier function that are associated with sepsis, hepatic steatosis, and injury [49]. Thus, enhancing intestinal mucosal barrier function and integrity, reducing mucosal immune overreactions, and improving gut microbes are the means to mitigate diseases related to IHDs.

However, our understanding of the regulatory molecular mechanisms underlying the development and progression of IHD-related diseases remains limited, particularly regarding their effects on the stability of the normal internal environment and the involvement of intestinal epithelial genes during disease. Additionally, the role of TFs with the potential to improve intestinal homeostasis in this process remains elusive. Therefore, ELF4 may become an attractive target and a new strategy for regulating IHDs or IHD-related diseases. In this paper, we aimed to elucidate the biological function of ELF4, with the hope of providing positive reference significance for gut-related diseases and MetS.

## 2. Methods

This review is based on articles retrieved from PubMed, Semantic Scholar, Web of Science, World Wide Science, and Embase using the following keywords: transcription factor ELF4, immune regulation, intestinal inflammatory-related disease, barrier function, gut microbiota, pathogen infection. In addition to a general search without year restrictions, a separate search was conducted for literature published between 2000 and 2025.

## 3. The Protein Structure of E26 Transformation-Specific (ETS)

**Figure 1:**
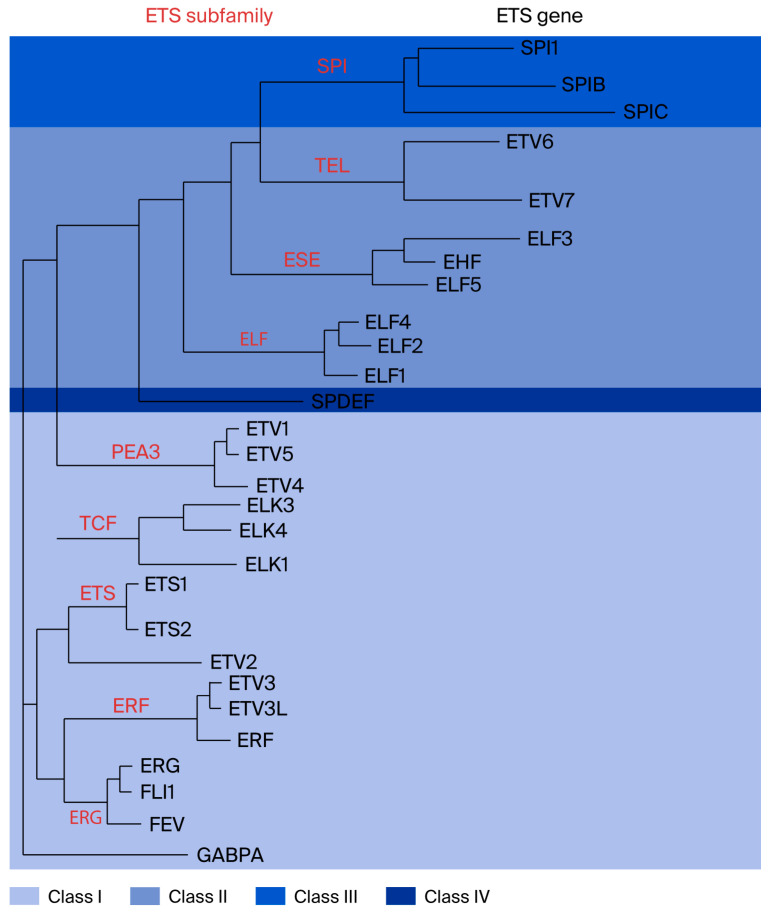
Classification of the ETS domain transcription factor family, with ETS family transcription factor ELF4. Red letters indicate ETS subfamily. Black letters represent ETS genes (Figure reproduced with permission from Hollenhorst et al.) [55].

The ETS is a type of TF that is evolutionarily conserved in multicellular organisms. In addition, the ETS TF family is one of the human body’s transcription regulator families (Figure 1). These family members all have a DNA-binding domain of approximately 85 amino acids (aa), called the ETS domain, which recognizes a stretch of 5′-GGA (A/T)-3′ or 5′-(A/T) TCC-3′ sequence to aid ETS family proteins in binding to the genomic region containing the target sequence [50,51]. The ETS domain-binding sequences are ubiquitous in a variety of megakaryocyte-specifically expressed genes [52]. An analysis of the binding sequences of all ETS family molecules has shown that although the physiological functions of the different ETS family proteins vary, the DNA sequences to which they bind have high similarity. The research suggests that the regulation of gene expression lies not only in binding with the target gene sequence but also in the exchange of information with other proteins or nucleic acids [53]. Ets-1 was the first member of the ETS domain TF family to be discovered. The names of Ets-1 and the entire family discovered afterward derive from the protein encoded by the v-ets oncogene found in the fusion protein expressed by avian myeloerythroblastosis virus E26 [54]. This family is divided into many subfamilies based on the sequence similarity of the ETS domain and the status of other important domains [55]. There are 28 members in humans and 27 in mice. The main functions of this family of proteins include the regulation of embryonic development, hematopoiesis, angiogenesis, nerve cell function, and participation in tumorigenesis [56,57], such as the pathogenesis and development of colon cancer [58]. Many ETS transcription factor family members are involved in the pathogenesis and development of cancers, mainly because these factors play important roles in basic physiological processes, such as cell growth, proliferation, and differentiation. They also have important regulatory functions in the immune system.

The E74-like factor (ELF) subfamily members include ELF1, ELF2/NERF, and ELF4/MEF, which regulate the immune response and immune cell development [59,60]. ELF3/ESE-1, ELF5/ESE-2, and ESE-3/EHF are members of another subfamily called epithelium-specific ETS (ESE). ELF1 was the first member of the ELF subfamily to be discovered and plays an important regulatory role in the maturation of erythrocytes and the development of NK cells, NKT cells, and T cells [60,61,62]. ELF2 may also be involved in tumor cell invasion and metastasis and vascular development [63].

ELF4 was first cloned in the megakaryocyte cell line in 1996, and it is a member of the ELF subfamily, a subfamily of the ETS TF family. ELF4 was named myeloid elf-1-like factor (MEF) because of its gene structure being similar to that of the earliest identified ELF family member, ELF1 [64,65]. Further analysis showed 94% homology with ELF1 in the ETS domain; thus, MEF was classified into the ELF family and named ELF4. However, although the DNA of ELF1 and ELF4 have high homology, the two genes regulate different targets [64], with only approximately 46% overlap [65]. ELF4 is located on the X chromosome (Xq26) and has high expression in multiple intestinal tissues and organs, with the most prominent high expression in the hematopoietic system, and it also exerts high expression in different developmental stages and different lineages [40,66]. This suggests that it may be involved in the regulation of immune cell development or function and the pathogenesis and development of hematological tumors.

**Figure 2:**
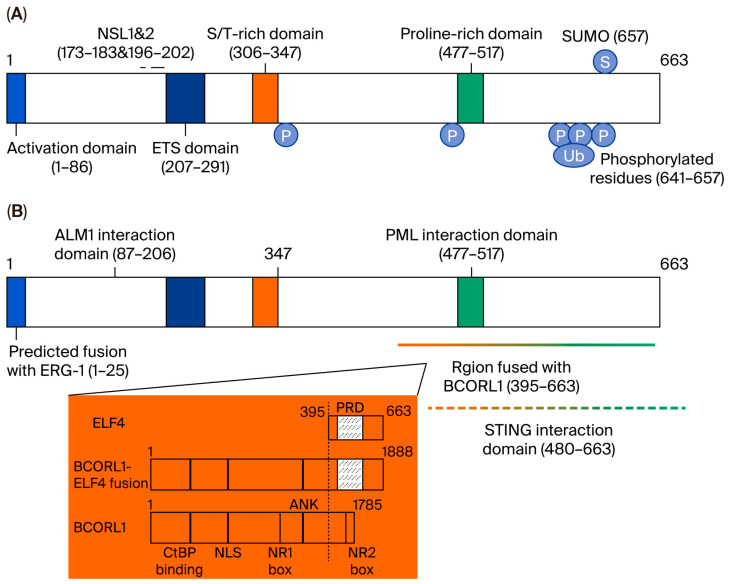
Protein structure of ELF4. (**A**) ELF4 has 663 amino acid residues, and the numbers in the diagrams indicate the amino acid residue sequence numbers. (**B**) ELF4 interacts with various proteins through different domains. AML1, PML, and STING interact with ELF4. Chromosomal translocation or inversion will lead to the protein fusion of ELF4 and another ETS transcription factor family member, either ERG1 or BCORL1 (Figure reproduced with permission from Suico et al.) [51].

ELF4 encodes a protein containing 663 aa (Figure 2), which contains an N-terminal activation domain, an ETS domain that binds to DNA sequences, a serine/threonine-rich region, and a proline-rich region [28,67]. Elf4 knockout did not cause side effects in physical growth development in mice, suggesting that it is nonessential to embryonic development or that other family members can compensate for its function. The ELF4 protein contains two nuclear localization sequences, which are located at aa sequences 173–183 and 196–202; the C-terminal serine and threonine residues of ELF4 can be phosphorylated. ELF4 can interact with proteins such as acute myeloid leukemia protein (AML1) or promyelocytic leukemia protein (PML) and stimulator of interferon genes (STING) through the N-terminus and C-terminus.

## 4. ELF4’s Biological Functions

ELF4 can and upregulate the transcription level of the human β-defensin 2 gene in epithelial cells [68,69]. Additionally, it is considered a potential tumor suppressor on the X chromosome [70]. At the basal level, ELF4 transcription is regulated by the transcription factor Sp1 [71], and its transactivation function is partly regulated by promyelocytic leukemia (PML) proteins [72]. Under the regulation of the small ubiquitin-related modifier (SUMO) protein, lysine 657 of human ELF4 is ubiquitinated, resulting in the inhibition of ELF4’s transcriptional transactivation ability on the lysozyme gene [73]. ELF4 is also involved in various physiological and pathological cell processes, such as tumorigenesis, the DNA damage response, and cell cycle regulation. Although there are an increasing number of studies in this field, the true biological function of ELF4 remains unclear [30,31,32].

### 4.1. Antiviral Innate Immunity of ELF4

ELF4 is a novel IFN-I TF. The absence of HIPK2 involved in ELF4 activation affects the host’s resistance to RNA viruses, emphasizing the function of ELF4 in antiviral innate immunity [28,74]. The innate immune system is the body’s first line of defense against invading pathogens, mainly by recognizing different conserved molecular motifs. This recognition method is called pathogen-associated molecular patterns (PAMPs). You et al. discovered that ELF4 is a type I IFN TF, i.e., this protein has an important role in the regulation of type I IFN responses and resistance to viral infections. This new progress is regarded as a breakthrough in the functional study of ELF4 [75]. Further quantitative analysis of mRNA and proteins has shown that ELF4 overexpression can induce the secretion of type I IFNs in 293T cells, and this process is mainly achieved through the interaction between the C-terminal region of ELF4 and STING. In addition, in vitro antiviral activity experiments have also shown that after stimulation with Sendai virus (SeV) or vesicular stomatitis virus (VSV) and interferon-β (IFN-β), the mRNA level and protein expression of ELF4 were significantly increased, and the resulting ELF4 overexpression can inhibit viral replication. In vivo experiments have shown that ELF4-deficient mice were more sensitive to virus infection after challenge with a lethal dose of West Nile virus (WNV) compared to WT mice, and the ability of macrophages to produce type I IFNs was damaged. This indicates that ELF4 is involved in IFN production and the body’s antiviral immune response. Early studies led to speculation that ELF4 plays an important role in the body’s antiviral cellular immune response [32,76]. You et al. confirmed that ELF4 inhibits viral replication by directly regulating the type I IFN response rather than inhibiting viral replication by activating NK cells or cytotoxic T cells [28].

**Figure 3:**
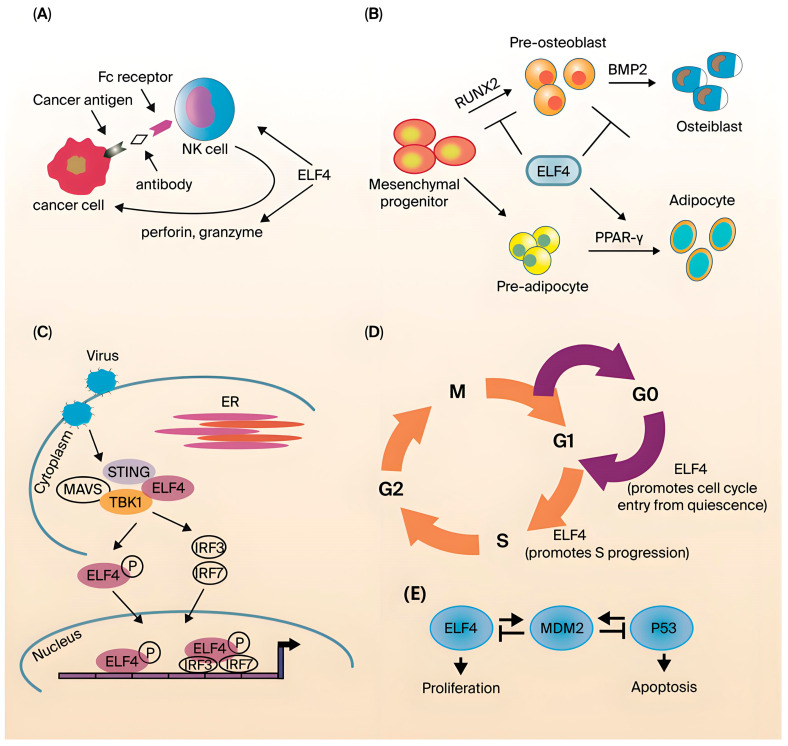
Biological function of ELF4. (**A**) ELF4 is needed for the development and function of NK cells. (**B**) During viral infection, ELF4 interacts with the immune signaling ligand STING to recruit and form the STING-MAVS-TBK1 complex. (**C**) ELF4 inhibits RUNX2-mediated and BMP2-dependent osteoblast differentiation, and ELF4 enhances PPAR-γ-induced adipocyte differentiation. (**D**) ELF4 drives hematopoietic stem cells from the G0 to the G1 phase and promotes cells from the G1 to the S phase. (**E**) ELF4 and P53 transcriptionally activate MDM2 (Figure reproduced with permission from Suico et al.) [51].

IFN production is an important event in the innate immune response. The early initiation mechanism is mediated by multiple factors, including pattern recognition receptors (PRRs), ligand proteins, kinases, and TFs. STING is an important transmembrane ligand protein, and its downstream activation occurs through TANK (TRAF family member-associated NF-kappa-B activator)-binding kinase 1 (TBK1). Transcription factors include IFN regulatory factor 3 (IRF3). TBK1 kinase is considered to be the body’s main immune weapon against viruses and intracellular infectious pathogens. The monitoring of PAMPs is mainly realized through intracellular toll-like receptors (TLRs), RIG-I-like receptors (RLRs), and several other nucleic acid sensors, such as AIM2-like receptors [77,78,79,80]. After the activation of PAMPs is induced, PRRs trigger NF-κB-dependent production of inflammatory cytokines, chemokine responses, and type I IFNs through the activation of IRF3 and IRF7. Type I IFNs are a key component for the host to establish an antiviral state and prevent virus replication in cells. Different ligand proteins are employed by PRRs to connect NF-κB and IRF TFs in downstream signaling pathways. The key element in these signaling pathways is adapter mitochondria antiviral signaling protein (MAVS), which is an ancient ligand protein that can assemble RLRs to NF-κB and initiate TBK1 and IRF3 signaling. The molecular mechanisms of the ELF4 regulation of type I IFNs have been documented. ELF4 not only participates in TLR, RLR, and dsDNA receptor-mediated signaling pathways but also activates MyD88, TRIF, STING, and MAVS. After that, ELF4 is phosphorylated via TBK1 and translocated into the nucleus, depending on STING and MAVS (Figure 3B). Subsequently, ELF4 can improve the binding efficiency of NF-κB, IRF3, and IRF7 to the promoters of various type I IFNs. In addition, ELF4 can promote the binding of IRF3 and IRF7 to the newly discovered type I IFN promoter enhancer element (EICE), thereby controlling and promoting the transcription of IFNs. In vivo experiments have confirmed that in *ELF4*^−/−^ mice, the efficiency of VSV-induced translocation of IRF3 and p65 to the IFN-βgene promoter and the translocation of IRF7 to the interferon alpha-4 (IFNα4) promoter are significantly reduced. The enhancer element EICE of the IFN-β gene promoter has a key role in supporting the interaction and exerting the synergic effect of ELF4, IRF3, and p65, thus indicating that ELF4 links the downstream components IRF3, IRF7, and p65 to the promoters of IFNs. In addition, similar to IRF7, the basal expression level of ELF4 in various tissues of the body is very low. This may be the body’s self-protection mechanism in the resting state, mainly to prevent unnecessary inflammation caused by high ELF4 expression levels. During pathogen invasion, ELF4 expression levels are rapidly upregulated to increase type I IFN expression levels to protect against pathogenic infection [81,82,83,84].

ELF4 has been identified as a key element in intracellular nucleic acid-sensitive PRR-mediated signaling pathways. This has opened new horizons for antiviral research and the study of innate immune response regulation. We can treat viral diseases by artificially increasing the body’s ELF4 levels to manipulate its IFN levels. However, this process involves the activation of innate immune receptors and the activation of related immune cells [85]. Additionally, the high expression of IFNs is likely to cause negative effects; that is, the artificially changed intensity of the IFN response may cause severe symptoms in the host or the body’s adaptive immune response [86,87].

Given the background dependence of ELF4-driven type I interferon signaling, its therapeutic strategy should focus on indirectly regulating upstream factors (such as SP1, HIPK2) to achieve precise activation and avoid immunopathological risks. The level or phosphorylation status of intestinal ELF4, the characteristics of blood ISG or the proportion of fecal Proteobacteria can be used as functional replacement indicators for ELF4. In patients with ELF4 deletion mutations, upstream targeted therapy may show the best efficacy, which provides a testable path for the precise treatment of ELF4.

### 4.2. ELF4 Resistance to Pathogenic Microorganism Infection

Given the role of ELF4 in the body’s antiviral innate immunity, it has been speculated that it also plays an important role in fighting pathogenic microbial infections. However, there are few reports about ELF4’s role in fighting pathogenic microbial infections in either human or animal models. Surprisingly, ELF4 does have an important role in fighting Plasmodium [88]. Malaria has been a major global health issue for centuries, especially in underdeveloped areas, and it poses a major threat to human health [89]. The traditional treatment for malaria entails artemisinin combination therapy (ACT) as the main component to remove Plasmodium from the body [90]. However, with the continuous emergence of drug-resistant strains, the effect of traditional ACT has become increasingly unsatisfactory. Therefore, identifying the mechanism of Plasmodium drug resistance, discovering new anti-Plasmodium drugs, and studying the body’s resistance to Plasmodium infection are of great significance to global Plasmodium infection eradication [91,92,93].

Although the function of the innate immune response in fighting Plasmodium infection has been reported, the key signal pathways are still unclear. Zhang et al. reported the function of ELF4 in the body’s resistance to Plasmodium infection and found a novel interferon-independent regulatory mode in the anti-Plasmodium innate immune response; that is, the antiviral innate immune signaling molecule ELF4 served as the transcription factor of PF4. The above studies provide a new perspective for the clinical treatment of malaria and other pathogenic microbial infectious diseases [88].

### 4.3. ELF4 Regulates Effects on Immune Cells

Functional studies of ELF4 have suggested that it has regulatory effects on the myeloid and T-cell line IL-3 and the activation of granulocyte macrophage colony-stimulating factor (GM-CSF) [64,65]. In hematopoietic cells, ELF4 can specifically regulate the transcription of IL-8 [94]. IL-8 is a chemokine secreted by a variety of cells, including macrophages, epithelial cells, and endothelial cells, and is important in the recruitment of inflammatory cells in inflammation [95].

ELF4 also exhibits regulatory effects on CD8^+^ T-cell function. Mamonkin et al. reported that a small number of OT-1 wild-type and OT-1 ELF4^−/−^CD8^+^ T cells were transplanted into wild-type mice either alone or together, suggesting that ELF4 can regulate the effector and memory produced in Lm-OVA infection, as well as the tissue distribution of CD8^+^ T cells, and this regulation is CD62L- and CXCR4-dependent. Abnormally high ELF4 expression can lead to an increase in memory CD62L^low^CD8^+^ cells in the spleen but causes no significant change in CD62L^hi^ cells. Therefore, ELF4 specifically promotes the formation of effector memory CD8^+^ cells. After ELF4-deficient mice were generated, the first observation was obvious developmental and functional defects in NK cells and NKT cells [96,97]. The number of ELF4-deficient NK cells and NKT cells declined, suggesting that cell development was defective (Figure 3A). However, the specific mechanism remains unclear. The functional defect can be attributed to the fact that ELF4 can bind to the promoter of perforin and is critical to the expression of basal levels of perforin [76]. The resting, proliferation, and homing of lymphocytes are critical to the immune system. With the in-depth study of ELF4 knockout mice, the function of ELF4 in T cells was also revealed. ELF4 is a key molecule that regulates the proliferation and homing of CD8^+^ T cells. That is, the direct activation of KLF4 expression achieves the negative regulation of CD8^+^ T-cell proliferation, and by inducing expression, ELF4 maintains the function of the memory T-cell surface molecules CD62L and CCR7. Additionally, the expression of CD62L and CCR7 achieved a regulatory effect on the lymph node homing of memory T cells [32,96,98]. In the experimental autoimmune encephalitis model, ELF4 knockout mice had significantly increased differentiation of Th17 cells, and IL-17 levels were also significantly upregulated. This finding suggested that the differentiation of CD4^+^ naive T cells into Th17 cells also requires the negative regulation of ELF4 [99]. Stewart et al. reported that a primary immunodeficiency disease suggested that ELF4 may also regulate B-cell function [100]. In a group of patients with X-linked hypogammaglobulinemia combined with growth hormone deficiency, the T mutation at position 1487 of the ELF4 gene was found to be C to C, resulting in a change in the amino acid at position 369 of this protein from serine to proline. Although the effect of this locus change on the function of ELF4 has not been reported, the authors speculated that the mutation of this locus may change the 3D structure of the protein or affect its interaction with other proteins [100]. As demonstrated by the findings above, ELF4 has important roles in both innate immunity and adaptive immunity.

### 4.4. The Roles of ELF4 in Development and Differentiation

Most ETS transcription factor family members are involved in the development and differentiation of tissues and cells, and ELF4 is no exception. In addition to affecting the development of NK and NK-T cells and the development of hematopoietic system-related cell lineages, ELF4 is also reported to affect bone cell development [76]. Kim et al. showed that in vitro experiments have confirmed that ELF4 is expressed at the highest level in MC3T3-E1 cells in the early stages of differentiation [101]. MC3T3-E1 cells are a mouse bone cell line that can differentiate into osteoblasts in vitro (Figure 3C). Osteoblasts and adipocytes are both derived from mesenchymal stem cells, and because they have the same progenitor cells, these cell lineages have a certain degree of plasticity. Further studies have shown that ELF4 interacts with RUNX2 to inhibit its binding to the osteocalcin promoter while repressing Dlx5 transcription and promoting Msx2 transcription. Dlx5 is involved in bone formation, and Msx2 is a negative regulator of osteoblast differentiation. Additionally, osteocytes and adipocytes have the same precursor cells [102,103]. Studies on ELF4 transgenic mice have shown that ELF4 overexpression results in fewer osteocytes and increased adipocyte differentiation. The increase in adipocytes may be due to the activation of PARPs by ELF4. In summary, ELF4 is involved in the differentiation of both osteocytes and adipocytes, but the specific signaling pathways need further study. In addition, there are currently no reports on ELF4 involvement in bone-related diseases such as osteoporosis.

Furthermore, ELF4 also plays critical roles in the cell cycle and proliferation [104]. Early ELF4-related studies showed that ELF4 promoted the transition of cells from G1 to S phase. Further studies indicated that when ELF4 is involved in this process, phosphorylation, ubiquitination, and finally degradation occur. However, the target genes regulated by ELF4 to achieve this function have not been revealed [105]. In mouse hematopoietic stem cells (HSCs), ELF4 also exerts a similar cell cycle regulatory function [106]. ELF4-deficient HSCs are mostly arrested in G0, suggesting that ELF4 can promote HSCs from the resting phase to the active phase (Figure 3D). Previous studies have suggested that ELF4 upregulates MDM2 expression (Figure 3E). MDM2 degrades p53, which is important for maintaining cells in a resting state, and p53 degradation eventually leads to the transition of cells into the active phase [107]. In addition, ELF4 promotes vascular endothelial cell entry into the active phase of cell division by upregulating CDK4 [108]. ELF4 can also bind to and transactivate peroxisome proliferator-activated receptor γ (PPARγ) to promote adipocyte differentiation. This reveals ELF4’s involvement in cell cycle regulation.

### 4.5. The Roles of ELF4 in DNA Damage Repair and Cancer

ELF4 has been indicated to contribute to the persistence of γH2AX DNA damage foci, leading to the promotion of the DNA damage response and induction of cell apoptosis. When ELF4 is lost, γ- irradiation can cause γH2AX DNA damage foci to quickly disappear, and DNA damage repair errors increase [31]. Du et al. showed that hydrogen peroxide induced more DNA damage in ELF4 knockout cells, and Elf4 knockout mice produced more colon tumors in an AOM-DSS colon cancer model [109]. The malignant phenotype of tumor cells is driven by changes in the expression of various TFs, including well-studied proteins such as p53 and Myc. Despite significant progress, little is known about several other TFs, including ELF4, and how they help form the carcinogenic process in cancer cells. Currently, the relevant role and molecular mechanisms of ELF4 in cancer development remain unclear; ELF4 exerts different functions in different tumors, and ELF4 has tumor-promoting effects in ovarian cancer and glioma [109,110]. However, relevant studies have shown that the absence of the neurogenic tumor suppressors miR-124, miR-128, and miR-137 is associated with the undifferentiated state of glioblastoma. Most of their effects come from the inhibition of oncogenic TF networks. Researchers performed high-throughput functional siRNA screens in glioblastoma cells and identified ELF4 as a major contributor to the oncogenic phenotype [111]. ELF4 knockout delayed proliferation and apoptosis of glioblastoma cells and resulted in long-term growth delay and morphological changes in glioma stem cells (GSCs). Additionally, ELF4 is critical to glioblastoma cell identity through the control of genes in two dependent pathways: RTK signaling (SRC, PTK2B, and TNK2) and lipid dynamics (LRP1, APOE, ABCA7, PLA2G6, and PITPNM2) [112].

In lung adenocarcinoma, plasma cell tumors, multiple myeloma, and colon cancer, ELF4 exerts a tumor suppressor function [70,109,113]. The earliest evidence that ELF4 is involved in cancer comes from its fusion protein with AML1 in acute myeloid leukemia [114]. This fusion results in the disappearance of the transcriptional activation function of ELF4, suggesting that ELF4 is critical to maintaining the normal differentiation of myeloid blood cells. The formation of the fusion protein of ELF4 and ERG-1 has also been found in acute myeloid leukemia, but the specific effect remains unclear [115]. In addition to its potential involvement in hematological tumors, ELF4 has also been reported to be involved in the pathogenesis and development of various solid tumors. For example, the BCORL1-ELF4 fusion transcript and protein were detected in a case of hepatitis C virus-positive hepatocellular carcinoma [116]. The fusion protein had an enormous functional impact on the two proteins, but it remains unclear how this effect is involved in the development of cancer. ELF4 is highly expressed in ovarian cancer and ovarian cancer cell lines, and cell malignancy declines after knocking down ELF4, suggesting that ELF4 is an oncogene in ovarian cancer. ELF4 is also highly expressed in gliomas. ELF4 knockout mice have impaired neurosphere formation and slow glioma growth, and ELF4 may promote the growth of gliomas by upregulating Sox2 [111]. In contrast to the abovementioned tumor-promoting effects of ELF4, other studies have revealed that ELF4 often functions as a tumor suppressor gene in epithelial tissues. Additionally, ELF4 expression is inhibited in various epithelial cancer cells, and ELF4 overexpression in the lung adenocarcinoma cell line A549 can inhibit the growth of cancer cells in nude mice. At the same time, in vitro studies have shown that the growth and invasion ability of ELF4-overexpressing A549 cells were both weakened [70]. These phenotypes may be due to the inhibition of MMP-9 and IL-8 expression via ELF4. Similar findings have also been reported for prostate cancer [117]. A recent study further confirmed that ELF4 functions as a tumor suppressor gene in various cancers. ELF4 overexpression in these cancer cell lines inhibits the proliferation of cancer cells, while the overexpression of the ELF4 mutant (E211M) does not have this effect because E211M loses its DNA-binding ability. Further genome-wide screening of the ELF4-binding locus has suggested that ELF4 can bind to the promoters of DLX3 and HRK, and these two genes have anti-proliferative abilities [118]. The above studies suggest that ELF4’s role in cancer is closely related to its tissue of origin and specific cellular context.

ELF4 has complicated physiological and pathological functions, and its expression must be tightly regulated. At the transcription level, the ELF4 promoter can be bound by SP1, E2F1, HIF1, and GFI1b, and its distal enhancer can be bound by PU.1, ERG, and FLI-1 [68,71,119,120]. The transcriptional repression of ELF4 by GFI1b is crucial to erythrocyte survival. In addition, the posttranscriptional protein levels of ELF4 are regulated by various modifications, such as ubiquitination by MDM2, the phosphorylation of CDK2 and TBK1, and modification by SUMO [51].

Advancements in multiomics and bioinformatics have helped to explicate how changes in ELF4 expression in human cancers are associated with disease outcomes and drug response in cancer cells. A study revealed that 32 different human cancers could be divided into tumors expressing high ELF4 transcripts and tumors expressing low ELF4, and patients in the two groups were associated with different clinical outcomes. In addition, tumors expressing high ELF4 mRNA levels tended to have higher grades, afflicted a significantly older patient population, and had significantly higher mutation burdens. Notably, there is a key point that the responses to 129 anticancer drugs in cell lines expressing high ELF4 mRNA transcription were significantly decreased, while the responses to three drugs, such as dasatinib, WH-4-023, and ponatinib, were significantly increased, all of which are significantly targeted by the proto-oncogenes tyrosine protein kinase SRC and tyrosine protein kinase ABL1. Based on this finding, new classifications of patient tumors, treatment, and prognosis strategies can be developed [30,121,122].

## 5. ELF4 Related Diseases

Based on the biological function of ELF4, we know that ELF4 could be involved in the maintenance of gut health and the development of diseases by targeting and regulating different genes across various cells, developmental stages, and pathological processes. Because of ELF4’s transcriptional regulatory role, ELF4 deficiency could lead to IHD-related diseases or MetS. For instance, a study reported that when nucleotide position 1487 of the ELF4 cDNA sequence is changed from T to C, serine at position 369 of the encoded protein will be replaced by proline. Any patient carrying this mutation will suffer from X-linked hypogammaglobulinemia associated with growth hormone XLH-GHD, a rare immune deficiency disease that manifests as chronic infection, tissue inflammation, and arthritis [123]. Additionally, the loss-of-function mutation of ELF4 can cause patients to develop symptoms of mucosal autoimmunity and IBD, manifested as low-grade fever and ulcers [33]. Next, we will discuss some important diseases related to ELF4 based on its biological functions.

### 5.1. Colorectal Cancer

Recurrent attacks of colitis are a predictive factor for colitis-associated colon cancer (CAC), and there is a strong positive correlation between the risk of CAC and the course, severity, and number of colitis attacks. Therefore, the promoting effect of inflammation on CAC seems self-evident. The mechanisms by which inflammation affects the pathogenesis and development of CAC have been the focus of previous studies, and these studies have indeed demonstrated the role of inflammation in the pathogenesis and development of CAC through various mechanisms. Gut microbiota imbalance is associated with a variety of diseases, such as obesity, inflammatory bowel disease, type 2 diabetes, metabolic syndrome, atherosclerosis, alcoholic liver disease (ALD), nonalcoholic fatty liver, liver cirrhosis, hepatocellular carcinoma, colon cancer [124,125,126], and even Alzheimer’s disease [127].

The interactions, influence, and regulation among the gut microbiota, inflammation, and host immune system have been shown, but in patients with ulcerative colitis, the causal relationship between the three remains unclear. The prerequisite for intestinal microbiota to induce inflammation is the destruction of the intestinal epithelial barrier and the recognition of microorganisms by the host immune system [128]. Based on this, it can be inferred that when intestinal microorganisms are disordered and the intestinal epithelial barrier is damaged, the initial contact between proinflammatory bacteria and host immune cells is likely to trigger a strong natural inflammatory response.

Du et al. found that gut microbiota-mediated downregulation of ELF4 increases the risk of ulcerative colitis complicated with colon cancer. In a dextran sodium sulfate-induced mouse chronic colitis model, it was unexpectedly found that *ELF4* gene-deficient mice were highly susceptible to colitis-associated colon cancer. Notably, ELF4 was significantly downregulated in the intestinal epithelial tissues of active ulcerative colitis patients and in mouse models. Notably, ELF4 has a protective effect on the epithelial barrier of the colon. Du et al. indicated that ELF4-deficient mice showed low expression of mucins and antimicrobial proteins accompanied by significant white blood cell infiltration, glandular fossa growth, and goblet cell loss. In addition, Du et al. also further reported that the proliferation of ILC3s in ELF4-deficient mice is impaired under *C. rodentium* infection, and the ability of cells to produce IL-22 is greatly reduced. Thus, the study suggests that the impaired function of ILC3s may also be one of the reasons for the fragile and easily disrupted intestinal barrier in ELF4-deficient mice [109].

### 5.2. Monogenic Autoinflammatory Diseases (mAIDs)

mAIDs are a group of heterogeneous diseases that mainly affect innate immunity and have various genetic causes. The genetic diagnosis of mAIDs can contribute to the management and treatment of patients [33,129,130]. However, a large number of sporadic and familial cases still do not have known genetic characteristics. In a report, Sun et al. recently identified ELF4 X-junction (DEX) deficiency as a novel mAID in pediatric patients with recurrent viral and bacterial respiratory infections, refractory oral ulcers, constipation, and arthritis. Sun et al. further identified a hemizygous variant in ELF4 (chrX:129205133 a > G, c.691 T > c, p. W231R) using whole-exome sequencing and revealed that mutated ELF4 could not bind to proteins with reduced expression, limiting viral replication and producing more proinflammatory cytokines [131]. Thus, the global DEX (ELF4-deficient, X-linked) patient population is small. Our understanding of this new disease, DEX, remains at a preliminary stage. Sun et al. conducted a multicenter cohort study of immune regulation disorders caused by the ELF4 variant in China. They described five cases, whose main manifestations were oral ulcers, inflammatory bowel disease-like symptoms, fevers of unexplained origin, anemia, and systemic lupus erythematosus. Furthermore, they identified a potentially pathogenic ELF4 variant in all cases. The pathogenicity of these variants was confirmed by detecting ELF4 expression in peripheral blood monocytes from patients and utilizing a simple IFN-b luciferase reporter gene assay [94].

### 5.3. Human Autoinflammatory Diseases: Interaction Between ELF4 and Early-Onset IBD

The TFs that limit the destructive potential of inflammatory immune cells remain elusive [33]. The discovery of pathogenic single-gene mutations in severe immune diseases in children provides a powerful and impartial perspective, through which physiological immune regulatory mechanisms can be directly elucidated in humans [132,133]. These diseases affect millions of patients, with an estimated prevalence of 1 in every 5000 people. To date, over 400 distinct single-gene congenital immune errors have been described. A recent study reported that loss-of-function variants of the X-linked ETS transcription factor gene *ELF4* were identified in patients with early-onset IBD and mucosal autoinflammation. Moreover, they also provided related evidence to the ELF4 mutant in cells from patients and newly generated mouse models. Considering their study, we found that ELF4 plays vital roles in disease, and it makes a significant contribution to the current understanding of DEX or IBD [134,135,136,137]. Moreover, ELF4 possesses extensive translational relevance to human inflammatory diseases in suppressing inflammation and preventing mucosal disease.

### 5.4. The Role of ELF4 in Intestinal Homeostasis, Mainly Barrier Function and Gut Microbiota

**Figure 4:**
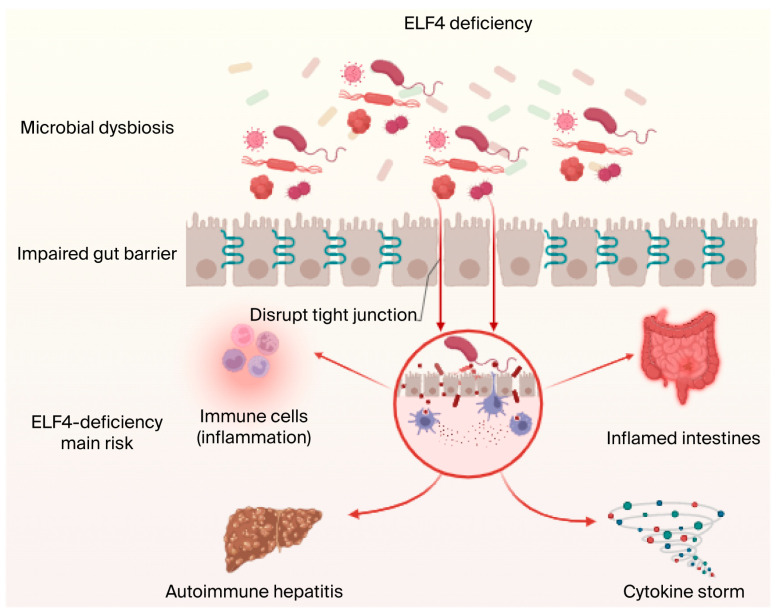
ELF4 knockout leads to inflammatory diseases, intestinal homeostasis disorder-related diseases, and metabolic disorders. ELF4 is related to many diseases. ELF4 deficiency or mutation can cause cytokine storms and pathological injury, further resulting in inflammation and mucosal disease. Additionally, ELF4 deficiency can lead to disruption of intestinal homeostasis, mainly including intestinal epithelial barrier disorder and gut microbiota imbalance, and cause susceptibility to IBD or ALD related to intestinal or MetS. Figure created in biorender https://biorender.com/.

The destruction of the gut microbiota and intestinal epithelial barrier is closely related to IHDs or MetS. Although various organizations, such as the World Health Organization, the International Diabetes Federation, and the American Academy of Clinical Endocrinology, have different definitions of MetS, these definitions all include central obesity, dyslipidemia, insulin resistance, and hypertension [138,139]. The term “metabolic syndrome” was proposed as early as 1970 [140], but it was not until 2007 that evidence for the gut-centered MetS theory emerged. A series of studies in rodents and humans showed that long-term consumption of a high-fat diet (HFD) can lead to intestinal barrier defects, which promote the development of intestinal luminal contents (including food antigens, bacteria themselves, and bacterial products, especially LPS) into the systemic circulation, resulting in metabolic endotoxemia [141,142]. Therefore, the study of the factors affecting intestinal permeability has become one of the most promising areas in microbiome research [143]. Moreover, both mouse models and patient studies have demonstrated that ELF4 loss of function can lead to enteritis (Figure 4), but the mechanisms underlying ELF4’s protective role in IBD and autoimmune diseases remain largely unknown. Additionally, intestinal epithelial cells and Paneth cells can maintain intestinal homeostasis through the production of mucin and antibacterial peptides [144,145], while intestinal barrier function impairment will damage the function of intestinal epithelial cells and Paneth cells, resulting in a leaky gut phenotype [146]. Moreover, leaky gut and intestinal flora disorders promote the development of various metabolic diseases, including ALD [144,147,148]. Therefore, exploration of the function of intestinal ELF4 deletion in intestinal homeostasis and the pathogenesis of ALD is warranted.

To explore the effect of ELF4 deletion on intestinal homeostasis under normal conditions using an ALD model, Liu et al. first found that antibacterial peptides and Muc 2 expression were downregulated in the intestinal tissues of *Vil1^Cre^Elf4^fl/fl^* mice, which in turn led to disorder of the intestinal microbiota, with a prominent increase in the proportion of Proteobacteria. In addition, they found that LPS levels in the feces of *Vil1^Cre^Elf4^fl/fl^* mice were the most important differentially abundant metabolite. Moreover, the LPS levels were positively correlated with the relative proportion of Proteobacteria. Additionally, elevated LPS reduced the levels of the tight junction proteins TJP1 and OCLN, thereby causing intestinal barrier damage [149].

ALD is the world’s most common liver disease and a leading cause of death worldwide. Intestinal barrier destruction and intestinal microorganism disorder are the typical characteristics of ALD [150,151,152,153]. The functional loss of ELF4 can lead to intestinal inflammatory disease, but the specific mechanism through which ELF4 affects intestinal homeostasis and the subsequent effects on the host remain unclear. Liu et al. further used an alcohol-induced mouse model of ALD to investigate the mechanisms of ELF4 in maintaining normal intestinal barrier function and intestinal homeostasis, as well as the protective effect on the host. As expected, ELF4 deficiency exacerbates alcohol-induced liver and intestinal inflammation and liver steatosis, cholesterol metabolism and lipid metabolism disorders, intestinal barrier dysfunction, and intestinal microbiota disorder. This study showed that ELF4 is an important host protection factor in epithelial cells that reduces IHD-related disease and MetS by maintaining the normal function of the intestinal barrier and intestinal microbiota homeostasis [149].

## 6. The Role and Significance of ELF4 Extend from Human Health to Overall Animal Health

Many monogastric animals, such as pigs, have gradually been widely used as an ideal model for studying human diseases [154]. They corroborate and complement human and medical research. In evolution, pigs have high homology with humans, and piglets’ gastrointestinal physiology and nutrient metabolism are similar to those of human infants. Therefore, piglets can be an ideal model for the study of infant gut physiology. Injury to intestinal mucosal barrier function can lead to enteropathy, such as enteritis and Crohn’s disease. Therefore, the study of the mechanism of protection of the intestinal mucosal barrier in neonatal piglets provides positive reference significance for the prevention and treatment of infant enteropathy. The enhancement of host intestinal innate immunity could be a crucial and feasible way to solve the global problem of bacterial resistance and reduce dependence on antibiotics. Animals, especially young monogastric animals (For example, piglets or weaned piglets) have immature intestinal tracts, as their intestinal microecological environment has not been established. They are especially susceptible to infection from pathogenic microorganisms such as Escherichia coli, Clostridium perfringens, and epidemic diarrhea viruses that cause intestinal disease, with mortality often exceeding 15% due to the influence of various stress factors [155]. The intestinal barrier function of weaned piglets is severely damaged, leading to reduced production performance or growth arrests, increased susceptibility to infections by various pathogenic microorganisms, and mortality rates of up to 10% [156,157]. Therefore, we suggest searching for alternatives to antibiotics that would promote piglets’ healthy growth and development and regulate intestinal homeostasis. Intestinal innate immunity plays an important role in resisting pathogenic microorganism infection. Additionally, intervening in the formation and/or reconstruction of the intestinal microbial flora structure of neonatal piglets and weaned pigs could improve the intestinal function of neonatal piglets and weaned pigs and reduce their risk of intestinal stress and pathogenic microorganism infection. ELF4 may become an attractive target and new strategy for regulating diseases related to intestinal homeostasis disorders. This could regulate piglets’ intestinal microbial homeostasis to relieve inflammatory injury. Therefore, in-depth research on its mechanism of action and taking it as a key target for antibiotic substitution can contribute greatly to human and animal health.

## 7. Conclusions

In the prevention of diseases related to IHDs or MetS, controlling inflammation was formerly the gold standard and formed the basis of most treatments. For instance, in ELF4-related studies, the downregulation of ELF4 is not entirely dependent on inflammation. Protection from intestinal microorgansism homeostasis can ensure the full expression of ELF4, which can more effectively repair various forms of DNA damage caused by inflammation. Therefore, when inflammation is controlled, disease prevention can be boosted by accounting for the gut microbiota. Based on the role of maintaining barrier function and gut microbiota homeostasis, especially during the period of inflammation, the activation of intestinal ELF4 may be a new strategy for disease prevention, but there is currently no agonist; its clinical use for prevention and treatment still needs in-depth exploration. In recent years, the supplementation of probiotics, prebiotics, and antibacterial peptides; the combined use of several probiotics; and the application of fecal microbiota transplantation have also played important roles in the treatment and improvement of diseases related to IHDs or MetS. Therefore, we can develop diets and drugs that activate intestinal ELF4 to prevent/treat intestinal diseases. We hope this paper provides new insight into intestinal ELF4 and other TFs as key protective factors, improving our understanding of the pathogenesis of diseases associated with intestinal homeostasis imbalance.

## Data Availability

No new data were created or analyzed in this study. Data sharing is not applicable.

## Abbreviations

The following abbreviations are used in this manuscript:

AA	amino acid
ACT	artemisinin combination therapy
ALD	alcoholic liver disease
AML1	acute myeloid leukemia protein
CAC	colitis-associated colon cancer
EICE	enhancer element
ELF4	E74-like Factor 4
ESE	epithelium-specific ETS
ETS	E26 transformation-specific
GLP-2	glucagon-like peptide-2
GM-CSF	granulocyte macrophage colony-stimulating factor
HFD	high-fat diet
HSCs	hematopoietic stem cells
HβD2	β-defensin 2
IBD	inflammatory disease
IFN	interferon
IHDs	intestinal homeostasis disorders
IRF3	IFN regulatory factor 3
KLB	Klotho
MAVS	mitochondria antiviral signaling
MEF	myeloid elf-1-like factor
MetS	metabolic disorders
PAMPs	pathogen-associated molecular patterns
PML	promyelocytic leukemia protein
PPARγ	peroxisome proliferator-activated receptor γ
PRRs	pattern recognition receptors
RLRs	RIG-I-like receptors
SeV	Sendai virus
STING	stimulate of interferon genes
TANK	TRAF family member-associated NF-kappa-B
TBK1	TANK-binding kinase 1
TF	transcription factor
TJPs	tight junction proteins
TLRs	toll-like receptors
VSV	vesicular stomatitis virus
WNV	West Nile virus
Xq26	X chromosome
